# The clinical behavior of mixed ductal/lobular carcinoma of the breast: a clinicopathologic analysis

**DOI:** 10.1186/1477-7819-8-51

**Published:** 2010-06-21

**Authors:** Aparna Suryadevara, Lakshmi P Paruchuri, Nassim Banisaeed, Gary Dunnington, Krishna A Rao

**Affiliations:** 1 Department of Internal Medicine, Southern Illinois University School of Medicine (SIU), Springfield, IL, USA; 2 Department of Pathology, Memorial Medical Center, 701 North First Street, Springfield, IL 62781, USA; 3 Department of Surgery, Southern Illinois University School of Medicine (SIU), Springfield, IL, USA; 4 Division of Hematology-Oncology, Department of Internal Medicine, Southern Illinois University School of Medicine (SIU), Springfield, IL, USA; 5 Simmons Cancer Institute at Southern Illinois University, Post Office Box 19677, Springfield, IL 62794-9677, USA

## Abstract

**Background:**

To date, the clinical presentation and prognosis of mixed ductal/lobular mammary carcinomas has not been well studied, and little is known about the outcome of this entity. Thus, best management practices remain undetermined due to a dearth of knowledge on this topic.

**Methods:**

In this paper, we present a clinicopathologic analysis of patients at our institution with this entity and compare them to age-matched controls with purely invasive ductal carcinoma (IDC) and historical data from patients with purely lobular carcinoma and also stain-available tumor specimens for E-cadherin. We have obtained 100 cases of ductal and 50 cases of mixed ductal/lobular breast carcinoma.

**Results:**

Clinically, the behavior of mixed ductal/lobular tumors seemed to demonstrate some important differences from their ductal counterparts, particularly a lower rate of metastatic spread but with a much higher rate of second primary breast cancers.

**Conclusions:**

Our data suggests that mixed ductal/lobular carcinomas are a distinct clinicopathologic entity incorporating some features of both lobular and ductal carcinomas and representing a pleomorphic variant of IDC.

## Background

Infiltrating ductal carcinoma is the most common type of invasive breast cancer, accounting for 65% to 80% of invasive breast lesions[1,2]. Its characteristics have been well described, including average age of onset, its rate of hormone receptor and erbB2 positivity, frequency of nodal involvement, rates of metastatic spread, and overall survival[3]. Historically, invasive lobular carcinomas (ILC) represented the second most common subtype of mammary neoplasia, accounting for about 5% to 10% of the disease[4]. The clinical behavior of ILC has been known to be different since its recognition as a distinct clinicopathologic entity[5]. Lobular carcinomas that are more frequently hormone-receptor positive[6] display a higher incidence of synchronous, contralateral primary tumors[7], more frequently present with multicentric disease[8], and metastasize to distinct sites such as the meninges, serosa, and retroperitoneum[9]. Given the difference in behavior between the two subtypes and the unique behavior of the ILC, the initial diagnostic workup has often involved the use of bilateral breast MRI to assess the state of the contralateral breast. The molecular characterization of breast cancer has substantially advanced with the categorization of mammary carcinomas into distinct molecular subtypes[10], and we now recognize the behavior patterns of breast carcinomas based on the molecular signatures that they bear[11]. However, this methodology has not yet become routine clinical practice. Fisher et al[12]. characterized over 1000 mammary carcinomas and recognized that the histologic subtypes could be mixed. They characterized approximately one-third of the lesions as invasive ductal carcinoma with one or more combined features. Slightly more than half of the combined tumors were IDC with a tubular component, and combinations with lobular carcinoma were detected in 6% of cases. It has also been seen that prognosis and survival of invasive breast carcinoma depends on the histology of the tumor[13,14].

More recently, with the advent of immunohistochemistry, it has been realized that one mixed histologic subtype of breast cancer, tubulolobular carcinoma of the breast, first described in 1977 by Fisher et al. represent a pleomorphic variant of ductal carcinoma. Tubulocarcinomas of the breast have classic grade I cytologic features and intimately mixed tubular and linear architecture[15]. The overall infiltrative pattern is that of lobular carcinoma, but the tumors are E-cadherin positive. Esposito et al. studied the clinical behavior of these tumors and concluded that the behavior of these tumors parallel their hybrid histology[16]. As E-cadherin was not lost in this tumor histology, the authors concluded that "It may thus be better termed 'ductal carcinoma, tubulolobular subtype', or 'ductal carcinoma with a tubulolobular pattern".

To date, the clinical presentation and prognosis of mixed ductal/lobular mammary carcinomas has not been well studied, and so little is known about the outcome of this entity. There is a trend of increased (about 2-fold increase) incidence of invasive ductal-lobular breast carcinoma from 1987 through 1999 in European studies, and Bharat et al.[17] describe an incidence of 6% in their US series[2,14]. To date, the best large study comes from Sastre-Garau et al[4]. They studied 11,036 patients with nonmetastatic breast cancer during the 1981-1991 period who were treated at the Institut Curie and prospectively registered in the Breast Cancer database. Among these patients, 726 cases corresponded to ILC, including the classical form and its histological variants, and 249 cases were classified as mixed ductal/lobular carcinoma. These two groups of ILC and mixed ductal/lobular carcinomawere compared with the group of 10,061 cases, mostly of the invasive ductal type (91% of cases), observed during the same period. The focus of the study was the comparison of ductal carcinomas to lobular carcinomas and predated the era of MRI imaging of the breast. Thus, best management practices remain undetermined due to a dearth of knowledge on this topic. In this paper, we present a clinicopathologic analysis of patients at our institution with this entity and compare them to age matched controls with purely invasive ductal carcinoma and historical data from patients with purely lobular carcinoma. We also perform E-cadherin staining to determine if this entity is best considered a variant of pleomorhic ductal carcinoma or a true lobular carcinoma.

## Methods

The study is a retrospective chart review of patients with invasive ductal and mixed ductal/lobular breast cancers diagnosed between 1990 and 1997. We obtained all cases (50) of mixed ductal/lobular breast carcinoma during that time period and matched them to 100 cases of IDC during the same time period. The cases of IDC were matched to the mixed ductal/lobular cases by both age and year of diagnosis to within one year. The data was obtained from the Cancer Registry at St. John's Hospital in Springfield, IL. Surgical pathology specimens of mixed ductal/lobular carcinoma were reviewed, and the percentage of lobular carcinoma was estimated as a percentage of the entire slide. The mixed ductal/lobular histology represents tumors in which "the ductal not otherwise specified (NOS) pattern comprises between 10% and 49% of the tumor, the rest being of a recognized lobular type" as defined by the WHO (Classification of tumours. Pathology and Genetics. Tumours of the breast and female genital organs. IARC Press, Lyon 2003). All slides were reviewed to generate a composite average for each case. Demographics and patient follow-up were obtained from the Cancer Registry at St. John's Hospital Cancer Institute. The variables evaluated include the patient's age at diagnosis, year of diagnosis, pathology, percentage of lobular and ductal in mixed carcinoma, type of surgery, tumor size, TNM stage, axillary lymph node involvement, adjuvant or neoadjuvant chemotherapy if given, radiation, multiple breast cancers, local and distant recurrences, overall survival, and disease-free survival. Overall survival, disease-free survival, and local recurrence were directly calculated from the data, and the association of clinical and pathologic variables was performed using Cox proportional hazards regression analysis.

Additionally, a list of all the patients who had MRI of the breast between years 2004 to 2007 was obtained from the Radiology Department at St. John's Hospital. After reviewing all the patients from the study group which included 100 cases of invasive ductal and 50 cases of mixed ductal/lobular pathology, we determined that a total of 23 patients had MRI of the breast. On further review of these pathology cases, there were 12 patients with mixed ductal/lobular carcinoma who had MRI of the breast. We then compared this group to 12 age-matched control patients with a diagnosis of IDC who had also undergone breast MRI at the time of initial diagnosis. Patients in the ductal group were additionally matched for the year of diagnosis to the mixed ductal/lobular histology group to within one year. We reviewed the MRI results of these cases and determined the rate of synchronous contralateral breast cancers in both groups and then performed a test of two proportions to determine if the rate of synchronous contralateral tumors significantly differed.

E-cadherin staining was done on the 7 cases of mixed ductal/lobular carcinomas as tissue blocks were available for these cases. Methods have been previously described[18-25]. Briefly, tissue was fixed in formalin, cut into 4 μm sections, dried overnight, and deparaffinized. Sections were rehydrated and underwent heat-induced epitope retrieval. Sections were stained using a Ventana Medical Systems (Tucson, AZ) automated system at 37°C for 32 minutes, followed by rinsing and cover-slipping. Immunohistochemistry was considered positive if staining was present above background levels. Known negative and positive controls were run with the samples.

Statistical analysis was performed on the data from both sets of patients. Logistic regression analysis using SPSS software was performed by holding the occurrence of a second primary breast cancer as a regression variable while the age at diagnosis, pathologic tumor grade, tumor size, and TNM stage were set as predictor variables. Cox regression analysis was further performed on the data set to determine if histology impacted overall survival or disease-free survival.

## Results

### Patient Demographics and Clinical Findings

All patients were female. Patients with mixed ductal/lobular tumors ranged from ages 32 to 90, with an average age of 62.28 years. Patients ranged in stages from I to IV. The majority of patients were stage I, with a percentage of 50% (25/50) presenting at this early stage. Stage II patients occurred at a frequency of 38% (19/50 cases), while stage III (3/50) and stage IV (2/50) comprised of 6% of cases and 4% of cases, respectively. Our control group of IDCs was comprised of 100 age-matched cases. The ages ranged from 31 to 90, with an average age of 59.07 years. The frequency of the various stages was stage I (48%), stage II (35%), stage III (15%), and stage IV (1%). Further patient characteristics are outlined in Table 1.

**Table 1:** Clinical characteristics

Clinical Characteristics	Invasive Ductal Carcinoma	Mixed Ductal/Lobular Carcinoma
Number of Cases	100	50

Age-Range (Year)	31 to 89	32 to 90

Age-Average (Year)	59.07	62.28

Stage I	48 (48%)	25 (50%)

State II	35 (35%)	19 (38%)

Stage III	15 (15%)	3 (6%)

Stage IV	1 (1%)	2 (4%)

Stage Unknown	1 (1%)	1 (2%)

Modified Radical Mastectomy	67 (67%)	31 (62%)

Partial Mastectomy	26 (26%)	14 (28%)

Simple Mastectomy	5 (5%)	5 (10%)

Unknown Specific Surgery	2 (2%)	0

Clinical characteristics of all the cases of invasive ductal carcinoma and mixed ductal/lobular carcinoma are summarized

Pathologically, 14% of the tumors in the mixed ductal/lobular group were grade I, 52% were grade II, 24% were grade III, and 10% were listed with an unknown grade. In the group of patients with invasive ductal carcinoma, 16% of patients had grade I tumors, 43% had grade II tumors, 30% had grade III tumors and 11% had an unknown grade. The average size of the primary tumors in the mixed ductal/lobular group was 22.1 mm with a range in size from 5 mm to 50 mm. In the ductal group, the average tumor size was 19.4 mm, with a range in size from 7 mm to 55 mm. This variable did differ significantly between the two groups when analyzed by ANOVA (p = 0.022). Axillary lymph nodes were involved in 26% of mixed ductal/lobular cases and 24% of ductal cases, respectively. Purely ductal carcinomas were 67% ER positive, 18% ER unknown, and 15% ER negative. They were 52% PR positive, 20% unknown, and 28% PR negative. Mixed ductal/lobular carcinomas were 74% ER positive, 10% ER unknown, and 16% ER negative. Mixed ductal/lobular carcinomas were 48% PR positive, 40% PR negative, and 12% PR unknown. Pathologic characteristics are outlined in Table 2.

**Table 2:** Pathological features

Pathological Features	Invasive Ductal Carcinoma	Mixed Ductal/Lobular Carcinoma
Number of Cases	100	50

Grade I	16 (16%)	7 (14%)

Grade II	43 (43%)	26 (52%)

Grade III	30 (30%)	12 (24%)

Grade Unknown	11 (11%)	5 (10%)

Tumor Size-Range	7-55 mm	5-50 mm

Tumor Size-Average	19.4 mm	22.1 mm

Axillary Lymph Nodes	24 (24%)	13 (26%)

ER Positive	67 (67%)	37 (74%)

ER Negative	15 (15%)	8 (16%)

ER Unknown	18 (18%)	5 (10%)

PR Positive	52 (52%)	24 (48%)

PR Negative	28 (28%)	20 (40%)

PR Unknown	20 (20%)	6 (12%)

Pathological features of all cases of invasive ductal carcinoma and mixed ductal/lobular carcinoma are summarized above. These include grade of tumor, tumor size-range and average, positivity of axillary lymph nodes and ER/PR status of the tumor.

**Figure 1:**
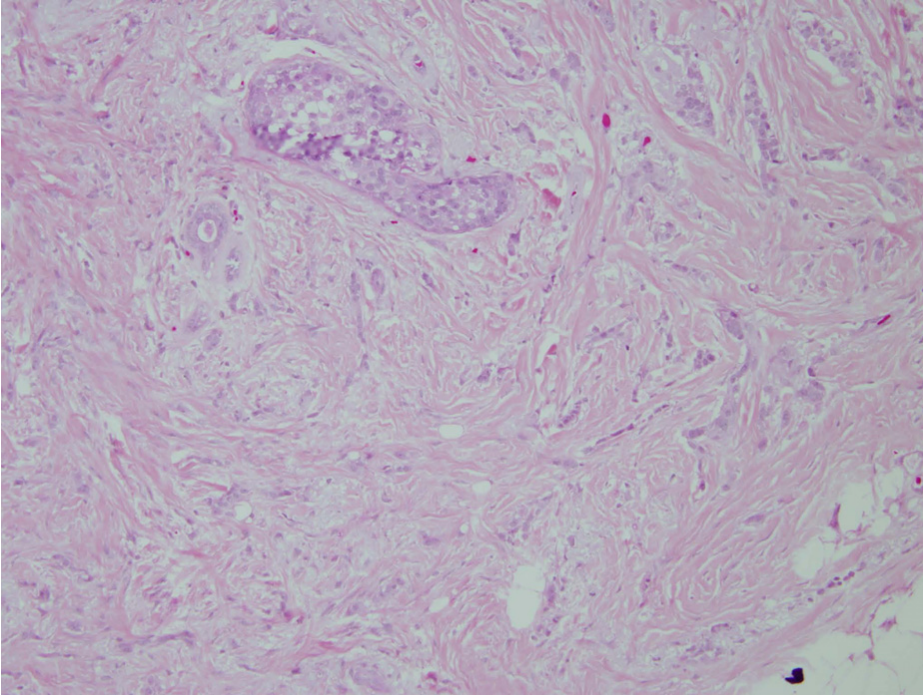
**H&E of a mixed ductal/lobular tumor**. 200× magnification of an H/E stained section of mixed ductal/lobular histology tumor. Ductal histology is noted.

**Figure 2:**
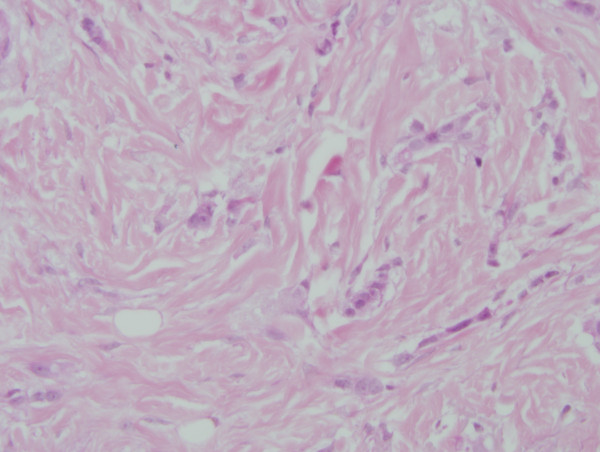
**H&E of a mixed ductal/lobular tumor**. 200× magnification of an H/E stained section of mixed ductal/lobular histology tumor. Lobular histology is noted.

**Figure 3:**
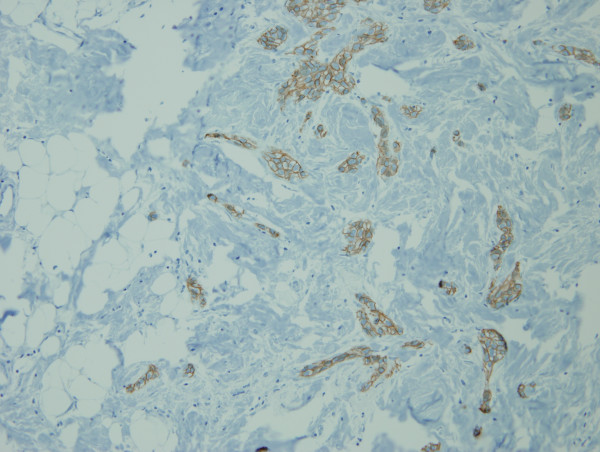
**E-cadherin immunostaining of the ductal components of the tumor**. E-cadherin staining on the same specimen displays the presence of E-cadherin in the ductal areas of the tumor.

**Figure 4:**
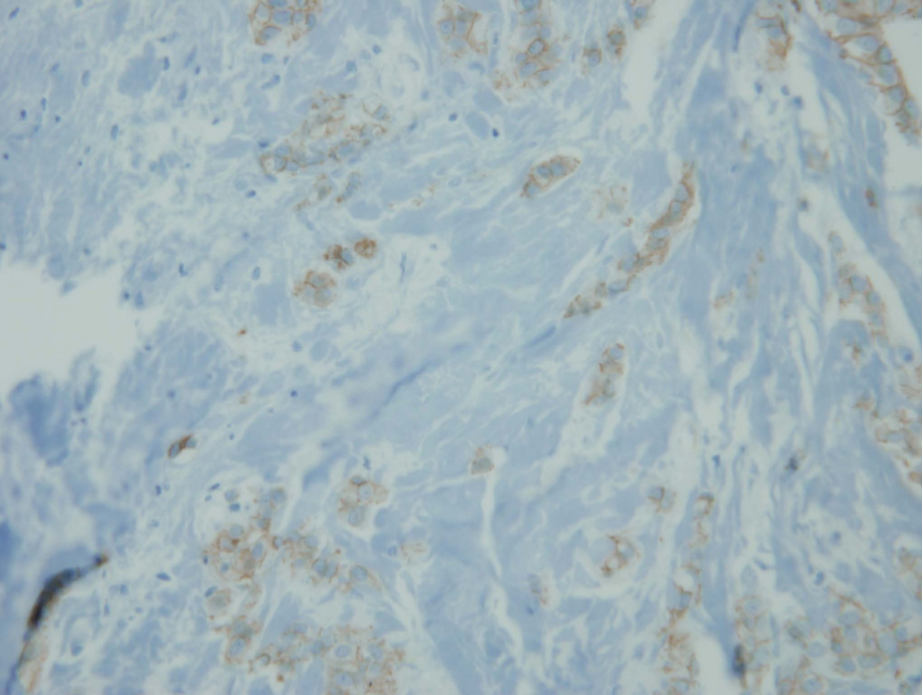
**E-cadherin immunostaining of the lobular components of the tumor**. E-cadherin staining on the same specimen displays the presence of E-cadherin in the lobular component of the tumor, suggesting that this mixed ductal/lobular tumor is indeed a variant of pleomorphic ductal carcinoma.

On review of the 7 available cases of mixed ductal/lobular carcinoma pathology, the average percentage of ductal carcinoma was 54.57% and the average percentage of lobular carcinoma was 45.28%. The individual percentage of lobular and ductal histology is listed for each case in Table 3. Figures 1 and 2 illustrate a case of mixed ductal/lobular histology, while Figures 3 and 4 illustrate the E-cadherin staining on that specimen, confirming E-cadherin presence in the ductal and lobular component of the tumor. Although the E-cadherin staining is weaker in the lobular regions of the tumor, it is nonetheless present. Overall, 90% (6/7) of the cases displayed E-cadherin positivity.

**Table 3:** Pathology slides

Case	Percentage of Ductal Carcinoma	Percentage of Lobular Carcinoma
1	22	77

2	70	30

3	50	50

4	60	40

5	50	50

6	80	20

7	50	50

Average	54.57	45.28

All the slides available (7 cases) on mixed ductal/lobular cases were reviewed under light microscopy and percentage of ductal and lobular carcinoma on each slide was estimated and summarized.

Post-surgical clinical treatment was subdivided into chemotherapy, radiation therapy, and hormonal therapy. Twenty-eight percent of the mixed ductal/lobular tumor patients and 30% of the ductal patients received chemotherapy. In the mixed ductal/lobular group receiving chemotherapy, 15.4% patients received doxorubicin, 30.8% received doxorubicin/cyclophosphamide, 38.4% received cyclophosphamide/methotrexate/5-FU, 7.7% received 5-FU/leucovorin/methotrexate, and 7.7% received methotrexate. In the invasive ductal group receiving chemotherapy, 3.7% received 5-FU, 11% received doxorubicin/cyclophosphamide, 26% received cyclophosphamide/doxorubicin/5-FU, 55.6% received cyclophosphamide/5-FU/methotrexate, and 3.7% received 5-FU/leucovorin/methotrexate. Thirty-two percent of the mixed ductal/lobular tumor patients and 31% of the IDC patients received radiation. Radiation consisted of 5040 cGy in 28 treatment fractions given to the whole breast followed by a 1000 cGy boost to the tumor in post-lumpectomy patients. Post-mastectomy patients requiring radiation received 5040 cGy in 28 treatment fraction to the involved chest wall. Hormonal therapy was given to 36% of patients in the mixed ductal/lobular histology group and 35% of patients in the purely ductal histology group. Virtually, all patients treated with hormonal therapy received tamoxifen in either group.

### Clinical Follow-up

Clinical follow-up was available for all patients and ranged from 10 to 17 years. In the mixed ductal/lobular group, the disease-free survival rate currently stands at 42% (21/50). Overall survival stands at 46%. Another 38% (19/50) patients have expired without any evidence of recurrence. Overall survival for this group averaged 12.2 years with a range of 10 to 17 years. Disease-free survival was an average of 8 years with a range of 0 to 15.25 years. Three patients were deemed "never disease-free", constituted 6% of the population, and have expired. An additional 6% (3/50) of patients had local or regional relapse of their tumor, and one of these patients has been successfully treated and remains disease-free. The rate of distant metastatic spread was 8% (4/50), and sites of metastasis included bone (2 patients) and lung (2 patients).

In the group of patients with ductal histology, the disease-free survival rate remains at 36%. Overall survival is 39%. Another 32% have expired without any evidence of recurrence. Eight percent of patients had a local or regional recurrence, 2% of patients had a recurrence that was not further specified, 3% were never disease-free, and 19% of patients developed distant metastatic spread. Liver metastases were present in 3% of patients. Lung metastases developed in 5% of patients. Bone metastases developed in another 11% of patients. Overall survival for the ductal group was 9.23 years with a range of 1 month to 17.8 years. Disease-free survival ranged from 0 months to 16.9 years with an average of 8.42 years. Interestingly, 30% (15/50) of the patients with mixed ductal/lobular histology breast cancer and 11% of the patients with ductal histology had a second primary breast cancer. There were 12 patients with mixed ductal/lobular breast carcinoma who had MRI of the breast, and 2 out of 12 patients (16.66%) had suspicious contralateral lesions. On review of the pathology, one case had carcinoma *in-situ *and other case had benign, fibrocystic changes in the contra-lateral breast. Thus, there was no case of contralateral invasive breast cancer. The 12 cases of invasive ductal breast carcinoma also had no contralateral breast cancer on review of the radiology results. One patient did have a benign fibroadenoma, but there were no cases of *in-situ *or invasive malignancy. Patient characteristics for these two groups are listed in Table 4.

**Table 4:** Clinical characteristics and MRI findings

Clinical Characteristics	Invasive Ductal Carcinoma	Mixed Ductal/Lobular Carcinoma
Number of Cases	12	12

Age-Range (Year)	37 to 78	33 to 84

Age-Average (Year)	53.2	54.5

Suspicious MRI findings	8% (1/12)	16% (2/12)

Invasive disease in contralateral breast	0%	0%

In situ disease in contralateral breast	0%	8% (1/12)

Other pathology in contralateral breast	8% (1/12- fibroadenoma)	8% (1/12- fibrocystic changes)

Clinical characteristics and pathologic findings in 24 patients undergoing breast MRI are outlined.

Statistical analysis was performed on the data from both sets of patients. Logistic regression analysis using SPSS software was performed. Holding the occurrence of a second primary breast cancer as a regression variable, age at diagnosis, pathologic tumor grade, tumor size, and TNM stage were not significant prognostic factors with β significance values of 0.447, 0.158, 0.490, and 0.424, respectively. However, the β value for tumor histology did achieve a significance of 0.05 and appears to be a strong predictor of secondary breast cancer development. The odds ratio of developing a second primary breast cancer was 7.95 with the mixed ductal/lobular histology using a Chi-square statistical model. Cox regression analysis was performed on the data set to determine if histology impacted overall survival or disease-free survival.

**Figure 5:**
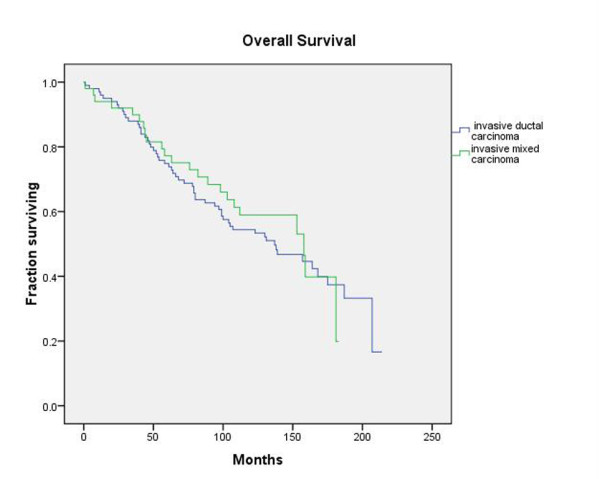
**Overall Survival**. Kaplan-Meier plot of overall survival of patients with mixed ductal/lobular histology tumors and patients with purely invasive ductal carcinomas reveals no significant difference between the two groups.

**Figure 6:**
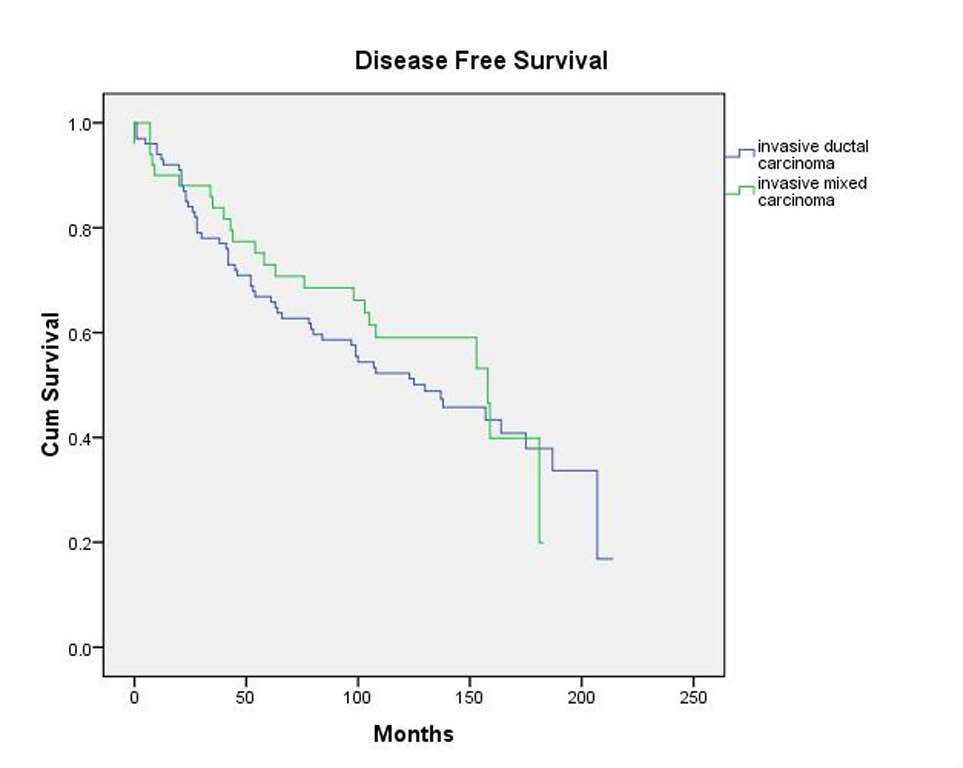
**Disease-Free Survival**. Kaplan-Meier plot of disease-free survival of patients with mixed ductal/lobular histology tumors and patients with purely invasive ductal carcinomas reveals no significant between the two groups as well.

Although TNM stage and tumor grade were both significant factors impacting overall survival and disease-free survival, histology was not a significant variable for both of these parameters, with a significance level of 0.728 with respect to overall survival and a significance level of 0.721 with respect to disease-free survival. Figure 5 depicts the overall survival curve for the two groups while Figure 6 depicts the disease-free survival for the two groups.

## Discussions

Analysis of our cases of mixed ductal/lobular histology has yielded several facts. Tumor size was larger in our mixed ductal/lobular histology patients, which may account for the higher rate of mastectomies. Tumor grade did not substantially differ between the ductal and mixed ductal/lobular histologies. Mixed ductal/lobular histology tumors were not significantly more likely to be ER negative and PR negative. Treatment did not also significantly differ between the 2 groups, with both groups receiving chemotherapy, radiation, and hormonal therapy at similar rates. Paired sample t-testing revealed no statistically significant difference between the 2 groups of patients (mixed ductal/lobular versus ductal histology) with regard to tumor grade or TNM stage.

Although the literature on this topic is scant, our data is in agreement with the published report of Sastre-Garau et al[4], who included 249 cases of mixed ductal/lobular histology tumors in their review. They noted a 10-year contralateral disease-free rate of 90% in ductal breast cancers and 90% in lobular cancers while mixed ductal/lobular histology tumors had a significantly diminished rate of 80%. As in our series, overall survival among the two subtypes did not significantly vary. In our more recently diagnosed cohort of patients, for whom breast MRI had become available, none of our patients had a contralateral synchronous invasive breast tumor and only one patient had a breast lesion (in the mixed ductal/lobular tumor group) which was pathologically a case of ductal carcinoma *in-situ *(DCIS). One patient in the IDC group had a benign fiboadenoma in the contralateral breast. Thus, there was no significant difference between the two groups of patients (mixed ductal/lobular histology and purely ductal carcinoma) with regard to synchronous contralateral breast tumors.

Arpino et al.[26] recently published their institutional experience with ILC. The median age of their patients with ILC was 64.6. The proportion of ER-positive tumors was 92.7%, and PR was expressed in 67.4% of ILCs. The pattern of metastatic dissemination in ILC was also different. ILC was three times more likely to metastasize to the peritoneum, gastrointestinal tract, and ovaries (6.7% versus 1.8%). Information on contralateral breast tumors was also available on the subset of 2,855 patients in whom sites of breast cancer distant from the primary could be assessed. Contralateral breast cancers in this group were more frequent among those with ILC (20.9%) than among those with IDC (11.2%). No difference in overall or disease-free survival was noted between ILC and IDC. Bharat et al. recently compared the outcomes for IDC, ILC, and mixed ductal/lobular histology tumors at their institution[17]. Patients with mixed ductal/lobular histology tumors and ILC were more likely to have low grade and hormone-receptor positive tumors, but also more likely to have stage III disease. The 10-year long-term survival, however, was better in patients with ILC (69%) and mixed ductal/lobular histology tumors (68%) than in patients with IDC (61%).

Clinically, in our series, the behavior of mixed ductal/lobular tumors seemed to demonstrate some important differences from their ductal counterparts. The rate of distant metastatic spread was much lower at 8% compared to a rate of 19% for the ductal tumors. The sites of spread were the lungs and bones, and mimicked the pattern of metastases by IDC. The rates of local/regional relapse were identical between the pure ductal and mixed ductal/lobular histology tumors, with both histologies demonstrating a 6% rate. However, the rate of second primary breast cancers was much higher at 30% compared with a rate of 11% with ductal histology. The rates of hormone-receptor positivity and age of onset were similar between the mixed ductal/lobular histology and purely ductal histology and did not differ from historically cited data in the literature[26]. However, unlike ILC, the rate of synchronous contralateral breast cancer (0%) was much lower, mimicking the ductal histology. This value was highly significant and suggests a different pattern of recurrence and different tumor biology. The literature also indicates that the mixed ductal/lobular histology, like ILC, is more likely to be associated with hormone replacement therapy[27,28]. Reeves et al. conducted 3.6 million person-years of follow-up on over 1 million post-menopausal women in the UK[28]. They found that the largest relative risks in current users of hormone therapy compared with never users of hormone therapy were seen for lobular (relative risk 2.25, 95% CI 2.00--2.52), mixed ductal/lobular (2.13, 1.68--2.70), and tubular cancers (2.66, 2.16--3.28). The only subtype that did not rise in incidence in their study was medullary carcinoma. Reeves et al. followed up with an analysis of reproductive factors and histologic subtype in their cohort of patients and interestingly found that "for most of the reproductive factors considered, the relative risks for mixed ductal/lobular carcinoma were intermediate between those found for ductal and lobular cancer[29]."

Our immunohistochemistry data suggests that mixed ductal/lobular carcinoma is another pleomorphic variant of IDC as 90% of the cases stained positively for E-cadherin. However, more tissue will be required to statistically confirm this trend. Of the available blocks, only one case did not stain for E-cadherin and may truly represent a lobular carcinoma. Wheeler et al.[30] have characterized tubulocarcinomas of the breast. Although our tumors did mostly stain for E-cadherin as well, we do not think our tumors represent tubulolobular carcinomas as "this histologic pattern is distinct from other mixed ductal/lobular carcinomas in which the invasive components are often separate and the tubular component lacks a lobular growth pattern." Additionally, morphologically, our cases did not meet the diagnostic criteria for tubulolobular carcinoma due to a lack of any tubular elements.

## Conclusions

Our data suggests that mixed ductal/lobular carcinomas are a distinct clinicopathologic entity incorporating some features of both lobular and ductal carcinomas and immunohistochemically may represent another variant of IDC. Scant information has been available to date on this entity. Based on our series of patients, it appears that routine breast MRI to screen the contralateral breast for an occult mammary malignancy is not warranted. However, clinical vigilance for the emergence of a second primary breast malignancy is mandated given the excessive rate of a second primary breast tumor.

## Competing interests

The authors declare that they have no competing interests.

## Authors' contributions

AS and LPP gathered and compiled data. NB served as pathologist and performed staining and interpretation of slides. GD first conceived of the study and provided valuable input on data evaluation. KAR performed statistical analysis and wrote the manuscript. All authors read and approved the final manuscript.
